# Brown tumors: Retrospective analysis of 26 cases

**DOI:** 10.1007/s00402-024-05372-9

**Published:** 2024-05-25

**Authors:** Mustafa Onur Karaca, Mustafa Özyıldıran, Merve Dursun Savran, Kerem Başarır, Hüseyin Yusuf Yıldız

**Affiliations:** 1 Department of Orthopedics and Traumatology, Ankara University, Ankara, Turkey; 2 Department of Orthopedics and Traumatology, Sandıklı State Hospital, Afyonkarahisar, Turkey; 3 Private orthopedic surgeon, Ankara, Turkey

**Keywords:** Brown tumor, Hyperparathyroidism, Parathyroidectomy, Intralesional curettage, Bone grafting

## Abstract

**Introduction:**

Brown tumors are reactive osteolytic lesions caused by hyperparathyroidism. These rare lesions are non-neoplastic processes that result from bone resorption. The purpose of this study was to retrospectively review a 34-year experience with brown tumors in our institution.

**Materials and methods:**

We retrospectively analyzed the records of 26 consecutive patients with brown tumor who were treated in our institution between May 1988 and October 2020, with a mean follow-up of 36,1 months.

**Results:**

17 male (65,4%) and 9 female (34,6%) patients with a mean age of 41,6 were included in the study. Localized bone pain was present in 13 cases (50,0%) as the first presenting symptom. 3 patients (11,5%) presented with diffuse bone pain. 7 patients (26,9%) were diagnosed with brown tumor while being investigated for pathological fractures. The other 3 patients (11,5%) were diagnosed while being evaluated for hypercalcemia symptoms. 7 patients (26,9%) had solitary lesions, while 19 patients (73,1%) had multiple lesions. Pelvis, femur, ribs, tibia, proximal humerus and mandible were the most common sites of localization. 23 patients (88,5%) were diagnosed with primary hyperparathyroidism, while the other 3 patients (11,5%) had secondary hyperparathyroidism. A total of the 65 lesions, 23 (35.4%) underwent orthopedic surgery, and 42 (64.6%) were followed up conservatively after parathyroidectomy. Orthopedic surgery was performed in 21 of 26 patients, the other 5 cases were followed up conservatively. Intralesional curettage was performed in 19 cases (82,6%). The resulting cavity was filled with bone cement in 11 cases (47,8%). Bone grafting was applied in 8 cases (34,8%). No recurrence was observed in any of the patients.

**Conclusion:**

The diagnosis of brown tumor begins with clinical suspicion. Endocrinology and general surgery consultation is important before surgery. Treatment of brown tumors requires a multidisciplinary approach.

## Introduction

Parathyroid hormone (PTH) is secreted by the parathyroid glands and regulates serum calcium concentration through its effects on bone, kidney, and intestine. Hyperparathyroidism (HPT) is an endocrine disease marked by increased secretion of parathyroid hormone, leading to imbalanced osteoclast activity [1–3]. Hyperparathyroidism can lead to nonneoplastic osteolytic lesions known as Brown tumors by affecting the bones. The brown tumor is also referred to as “osteitis fibrosa cystica” [4, 5]. The characteristic features of the lesion include non-neoplastic reactive tissue, extensive bone resorption, presence of osteoclasts with multinucleated giant cells, vascular and proliferating fibrous tissue, and the presence of hemorrhage. The term “brown tumor” is derived from its color, which is due to its rich vascularity, hemorrhage, and deposits of hemosiderin [5, 6].

Brown tumors are reactive osteolytic lesions caused by primary, secondary or tertiary hyperparathyroidism [1, 2, 4]. Primary hyperparathyroidism (PHPT) is a disorder caused by excessive secretion of PTH. The most common causes of primary hyperparathyroidism are parathyroid adenoma (80%) and parathyroid hyperplasia (10–25%). Multiple adenomas (5%) and parathyroid carcinoma (< 1%) are rarely seen. Hypercalcemia and hypophosphatemia are the most common laboratory findings in PHPT [1–4].

Secondary hyperparathyroidism (SHPT) is a common complication of chronic renal failure. In late stage kidney diseases, the synthesis of calcitriol, which is the active form of vitamin D, is impaired and hypocalcemia is observed. Phosphate excretion is also impaired. Decreased ionized calcium level and elevated phosphate level lead to continuous stimulation of the parathyroid glands, resulting in increased release of PTH [1, 7, 8]. Tertiary hyperparathyroidism (THPT) is a condition that occurs when secondary hyperparathyroidism persists for an extended period and the parathyroid glands become autonomously overactive. Tertiary hyperparathyroidism occurs as a result of end stage renal disease, just like SHPT [1, 7].

The incidence of brown tumor is reported to be approximately 3-4.5% in primary hyperparathyroidism and 1.5-2% in secondary hyperparathyroidism in the literature [4, 8, 9]. In recent years, thanks to improved screening techniques, hyperparathyroidism can be diagnosed at an earlier stage. Early detection and treatment of hyperparathyroidism have led to a decrease in the incidence of brown tumor, which is a classic manifestation of hyperparathyroidism [10, 11].

Radiographically, brown tumors are observed as well-defined expansile lytic lesions with a thin peripheral bone shell. Osteopenia, subperiosteal bone resorption, and the salt and pepper sign in the skull are other radiographic findings that can accompany brown tumors in hyperparathyroidism [4, 11]. Brown tumors can be observed as solitary or multiple lesions. Multiple bone lesions may be misdiagnosed in radiological imaging as bone metastases. In the differential diagnosis, biochemical analyses and the measurement of PTH have an important role [4, 11, 12].

Brown tumors are commonly observed in the pelvis, ribs, clavicle, mandible, femur, and other long bones. Various clinical signs and symptoms may be observed depending on the size and location of the lesions. Localized bone pain, diffuse skeletal pain or pathologic fracture can be observed. On the other hand, some patients remain asymptomatic and receive an incidental diagnosis through radiography [1, 4, 5, 8, 11].

The treatment methods for brown tumors are determined by the size and location of the lesions as well as the functional issues associated with them. The initial stage of the treatment involves controlling hyperparathyroidism and adjusting calcium, phosphate blood levels in patients. For some lesions, parathyroidectomy is sufficient, and the lesions may regress with recovery of imbalanced osteoclastic activity. On the other hand, conservative treatment is not sufficient for some lesions, and orthopedic surgeries are applied to prevent the risk of pathological fractures and to achieve better functional results [4, 8, 11, 13].

Brown tumors are among the rare entities that need to be remembered in the differential diagnosis when dealing with bone lesions. Clinical suspicion is important for the diagnosis, and there is no consensus for the treatment algorithm. Treatment planning should be made according to the patient and the lesion. The aim of this research was to retrospectively review a 34-year experience with brown tumors in our institution.

## Materials and methods

We retrospectively analyzed the records of 26 consecutive patients with brown tumor who had been treated in our institution during the period from May 1988 to October 2020. A total of 26 patients were included in the study, consisting of 24 patients who received their initial treatment at our institution after diagnosis and 2 patients who were referred to our clinic after parathyroidectomy.

We examined the files in the hospital archive of all patients who received treatment with the diagnosis of brown tumor within the specified time frame. The consultation notes of endocrinology and general surgery were evaluated using the hospital archive, pathology reports were examined, biochemical results (Ca, P, PTH, ALP) were reviewed. All patients whose diagnosis of brown tumor was histologically confirmed according to the pathology report and whose laboratory results were consistent with hyperparathyroidism were included in the study.

**Fig. 1:**
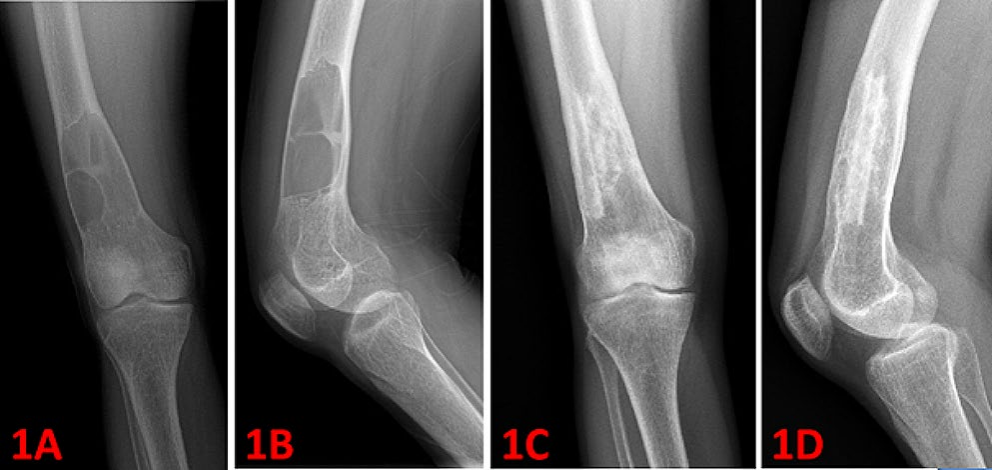
A 18-year-old female with osteolytic lesion (brown tumor) in the right distal femur (1 A and 1B: preoperative radiograms). Curettage was performed, followed by iliac crest autografting and cancellous allografting was applied. Figure 1 C and 1D shows postoperative 12th months radiograms

The preoperative radiological images of the patients were examined. Preoperative plane radiographs of all patients (26 patients) were obtained from hospital records. CT views were available for 17 patients, while MRI images were available for 15 patients. Scintigraphy images of 11 patients were available. By evaluating radiological images, data regarding parameters such as the size, number and location of the lesion were recorded.

The patients were classified according to the types of hyperparathyroidism. It was determined which patients underwent parathyroidectomy. The lesions were classified as those undergoing orthopedic surgical procedures or those followed conservatively. Surgical procedures were categorized by examining the operation reports in hospital records. Postoperative complications such as wound problems and implant failure were investigated by examining postoperative radiographic images.

**Fig. 2:**
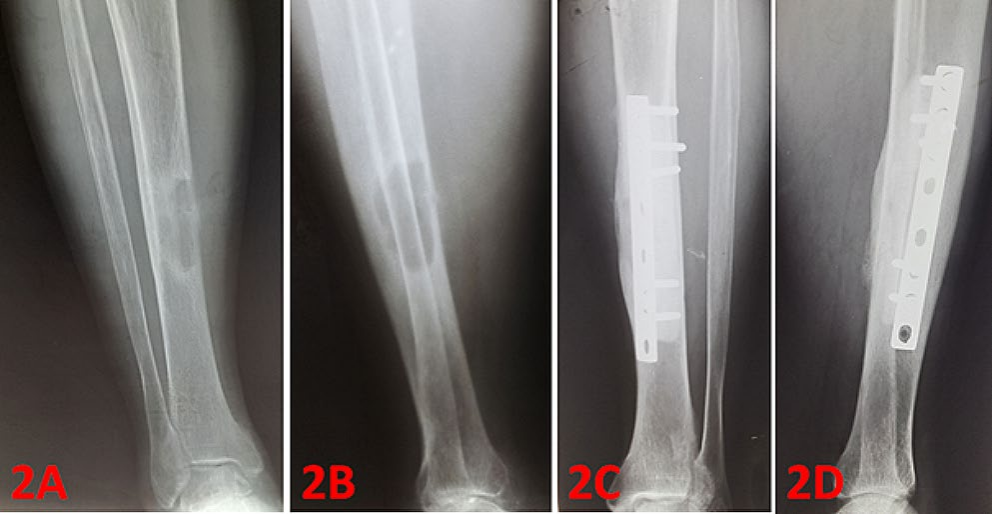
A 47-year-old male with brown tumor in the right tibial diaphysis (2 A and 2B: preoperative radiograms). Curettage and cementing was applied, followed by fixation was perfomed by using DCP plate. Figure 2 C and 2D shows postoperative 48th months radiograms

Statistical analysis was performed using SPSS v.22.0 (SPSS Inc.,Chicago, IL). Descriptive statistics were used to characterize demographic variables of patients. Mean, median and standard deviation values were used to show descriptive statistics.

## Results

17 male (65,4%) and 9 female (34,6%) patients with a mean age of 41,6 were included in the study. Mean follow up period was 36,1 months. Localized bone pain was present in 13 cases (50,0%) as the first presenting symptom. 3 patients (11,5%) presented with diffuse bone pain. 7 patients (26,9%) were diagnosed with brown tumor while being investigated for pathological fractures. The other 3 patients (11,5%) were diagnosed while being evaluated for hypercalcemia symptoms such as fatigue, headache, nausea, vomiting, constipation, abdominal pain (Table 1).

**Table 1:** Demographic variables of patients

Age		
Mean	41,62	
Median	44	
Min-Max	(18–68)	
Standard deviation (SD)	12,28	
Gender (n,%)		
Male	17	65,4%
Female	9	34,6%
Follow up period (months)		
Mean	36,04	
Median	27	
Min-Max	(12–156)	
Standard deviation (SD)	36,0	
First presenting symptom (n,%)		
Pathologic fracture	7	26,9%
Widespread pain	3	11,5%
Localized pain	13	50,0%
Hypercalcemia symptoms	3	11,5%
Number of lesions (n,%)		
Solitary lesion	7	26,9%
Multiple lesions	19	73,1%
Types of hyperparathyroidism (n,%)		
Primary	23	88,5%
Secondary	3	11,5%
Types of treatment (n,%)		
Conservative	5	19,2%
Surgery	21	80,8%

23 patients (88,5%) were diagnosed with primary hyperparathyroidism, while the other 3 patients (11,5%) had secondary hyperparathyroidism **(**Table 1**)**. Patients diagnosed with hyperparathyroidism were consulted to the departments of endocrinology and general surgery. All 23 patients diagnosed with primary hyperparathyroidism underwent parathyroidectomy by general surgery. Secondary hyperparathyroidism was observed in 3 patients due to chronic kidney disease. Parathyroidectomy was not performed in these three patients and medical treatment was applied by the endocrinology department.

The most common sites of localization were the pelvis, femur, ribs, tibia, proximal humerus, and mandible (Table 2). 7 patients (26,9%) had solitary lesions, while 19 patients (73,1%) had multiple lesions. A total of 65 lesions were observed in 26 patients. Orthopedic surgery was performed in 21 of 26 patients, the other 5 cases were followed up conservatively. Among the 65 lesions, 23 lesions (35,4%) underwent surgery, whereas 42 lesions (64,6%) were followed conservatively (Table 2). Intralesional curettage was performed in 19 cases (82,6%). The resulting cavity was filled with bone cement in 11 cases (47,8%). Bone grafting was applied in 8 cases (34,8%) (see Figs. 1, 2; Table 3).

**Table 2:** Anatomical locations and types of treatments

Location	Total number (*n*,%)	Orthopedic surgery (*n*,%)	Conservative treatment (*n*,%)
Iliac crest	9 (13,8%)	5 (55,6%)	4 ( 44,4%)
Femur diaphysis	7 (10,8%)	4 (57,1%)	3 (42,9%)
Ribs	5 (7,7%)	0	5 (100%)
Proximal femur	5 (7,7%)	3 (60,0%)	2 (40,0%)
Tibia diaphysis	4 (6,2%)	2 (50,0%)	2 (50,0%)
Proximal humerus	4 (6,2%)	0	4 (100%)
Acetabulum	4 (6,2%)	0	4 (100%)
Mandible	3 (4,6%)	0	3 (100%)
Proximal tibia	3 (4,6%)	0	3(100%)
Distal femur	3 (4,6%)	2 (66,7%)	1 (33,3%)
Phalanx	3 (4,6%)	1 (33,3%)	2 (66,7%)
Clavicle	2 (3,1%)	0	2 (100%)
Ischium	2 (3,1%)	0	2 (100%)
Scapula	2 (3,1%)	0	2 (100%)
Fibula diaphysis	2 (3,1%)	2 (100%)	0
Lumbar vertebrae	1 (1,5%)	0	1 (100%)
Thoracic vertebrae	1 (1,5%)	1 (100%)	0
Sacrum	1 (1,5%)	0	1 (100%)
Distal radius	1 (1,5%)	1 (100%)	0
Sternum	1 (1,5%)	0	1 (100%)
Metacarp	1 (1,5%)	1 (100%)	0
Metatars	1 (1,5%)	1 (100%)	0
*Total*	*65 (100%)*	*23 (35,4%)*	*42 (64,6%)*

**Table 3:** Treatment methods

Treatment methods	(N)	%
Curettage + cement	4	17,4
Curettage + autograft	4	17,4
Curettage + cement + Kuntscher nail	4	17,4
Curettage + cauterization + high speed burr + cement + plate fixation	2	8,7
Curettage + cauterization + cement	1	4,3
Curettage + cauterization + autograft	1	4,3
Curettage + cauterization + high speed burr + allograft + plate fixation	1	4,3
Curettage + cauterization + high speed burr + allograft + intramedullary nail	1	4,3
Curettage + autograft + plate fixation	1	4,3
Anterior thoracic fusion	1	4,3
Total hip arthroplasty	1	4,3
Resection	1	4,3
Ray amputation	1	4,3
*Total*	*23*	*100*

Pathological fractures were observed in 7 of 26 patients in our series. Three of these fractures were in the diaphysis of the femur, two was in the proximal humerus, one was in the neck of the femur, and one was in the distal radius. Two patients with pathological proximal humerus fractures were followed up with a velpeau bandage after parathyroidectomy. Union was achieved with conservative treatment without the need for orthopedic surgery. In the other 5 patients, orthopedic surgeries were performed due to pathological fractures. Among the three patients with femoral diaphysis fractures, orthopedic surgery was performed prior to the parathyroidectomy procedure in two of them. In the other patient with a femoral diaphysis fracture, after the parathyroidectomy procedure was performed and calcium-phosphate balance was restored, orthopedic surgery was carried out. A patient with a brown tumor in the femoral neck region underwent parathyroidectomy in an outside institution and was followed up conservatively. Three months after the parathyroidectomy procedure, the patient was referred to our clinic due to a pathological femur neck fracture, and total hip arthroplasty was performed (Table 4).

**Table 4:** Pathological fractures due to brown tumors and treatment methods

Case no	Age	Gender	Location	Parathyroidectomy	Orthopedic treatment
1	29	Female	Femur diaphysis	Parathyroidectomy was performed prior to orthopedic surgery	Curettage + cement + Kuntscher nail
2	27	Male	Proximal humerus	Conservative treatment was applied after parathyroidectomy	Conservative
3	52	Female	Femur diaphysis	Orthopedic surgery was applied before the parathyroidectomy	Curettage + cement + Kuntscher nail
4	38	Female	Proximal humerus	Conservative treatment was applied after parathyroidectomy	Conservative
5	44	Male	Femur neck	Parathyroidectomy was performed prior to orthopedic surgery	Total hip arthroplasty
6	32	Female	Distal radius	Parathyroidectomy was performed prior to orthopedic surgery	Curettage + autograft
7	40	Male	Femur diaphysis	Orthopedic surgery was applied before the parathyroidectomy	Curettage + cement + Kuntscher nail

Postoperative complications such as wound site problems or implant failure were not observed in patients who underwent orthopedic surgery. No recurrence was observed after orthopedic surgery. It was observed that the lesions of 5 patients who were followed conservatively had regressed after parathyroidectomy.

## Discussion

Osteitis fibrosa cystica was first described by Recklinghausen in 1891 [8, 14]. It is referred to as “brown tumor” in the literature due to its color resulting from rich vascularity and deposits of hemosiderin [1–3, 5, 6]. Primary, secondary, or tertiary hyperparathyroidism can cause brown tumors [15–20]. The clinical presentation of primary hyperparathyroidism has undergone significant changes over the past several decades. It is known that classic overt complications such as brown tumors, nephrolithiasis, and cholelithiasis are now seen less frequently. In older literature, the prevalence of brown tumors in PHPT was reported as high as 58–69%, whereas in present times, especially in developed Western countries, this rate is observed to be under 5% [5, 10, 11, 21]. In developed countries, routine serum calcium screening started to be applied in the early 1970s. This situation has facilitated the early diagnosis of PHPT in asymptomatic cases. Severe forms of PHPT accompanied by brown tumor have become rare with the widespread use of biochemical screening tests. One possible explanation for the decreased frequency of brown tumors in western industrialized countries could be related to less severe vitamin D deficiency. It is known that severe skeletal manifestations like brown tumors can still be frequently observed in countries such as China, India, and Thailand where severe vitamin D deficiency persists [10, 22, 23]. Vitamin D deficiency is also common in our country and is an important public health problem [24, 25]. This may have increased the number of patients admitted to our clinic with the diagnosis of brown tumor.

Brown tumors can be observed as solitary or multiple lesions. In the differential diagnosis of a solitary bone lesion of brown tumor, other potential causes such as aneurysmal bone cyst, solitary bone cyst, giant cell tumor, and giant cell granuloma should also be considered. Multiple bone lesions of brown tumor may be difficult to differentiate from osteolytic metastases, multiple myeloma, Langerhans’ cell histiocytosis, and leukemia. Brown tumors are non-neoplastic lesions [4, 11, 26, 27]. Their histopathology is characterized by multinucleated giant cells, proliferating fibrous tissue, and brown hemosiderin deposition [5, 6]. Histology alone cannot provide a definitive diagnosis, as other lesions, such as giant cell tumors, giant cell granulomas, and aneurysmal bone cysts, may exhibit similar macroscopic and microscopic characteristics [4, 11, 25, 26]. Biochemical analyses and the measurement of PTH level play a crucial role in establishing a definitive diagnosis [4, 11, 12].

Multiple bone lesions of brown tumor can imitate bone metastases in radiological imaging. The distinction between brown tumors and metastases can be readily made by measuring PTH levels. In metastases, hypercalcemias of malignancy can be observed, but elevations of PTH are almost never seen. On the other hand, the PTH levels are elevated in brown tumors [4, 8, 11, 27].

Clinical suspicion is important for the accurate diagnosis. Brown tumors should also be kept in mind while evaluating bone lesions, otherwise a misdiagnosis may occur. In the case series by Misiorowski et al., they reported three cases of brown tumors in which patients presented with pathological fractures and were initially misdiagnosed as having malignant tumors. In one of the mentioned cases, amputation was initially planned due to a biopsy in the proximal tibia that was consistent with osteogenic osteosarcoma. However, upon reevaluation of the biopsy results and laboratory findings, a diagnosis of Brown tumor was established. In the other two cases, multiple lesions were initially misdiagnosed as metastases, hypercalcemia was evaluated as humoral hypercalcemia of malignacy (HHM), and erroneous palliative radiotherapy and chemotherapy were administered [11]. De Crea et al. reported three cases initially misdiagnosed as malignancy in a series of 615 patients who underwent parathyroidectomy for PHPT. Two of these patients underwent resection due to misdiagnosis, and one underwent transtibial amputation [21].

The primary treatment for brown tumors is the appropriate management of hyperparathyroidism. The first step in treatment involves controlling hyperparathyroidism and regulating calcium and phosphate levels in patients. Parathyroidectomy is an adequate treatment for some lesions, and the brown tumors usually regress after parathyroidectomy. On the other hand, parathyroidectomy may not be sufficient for some lesions and orthopedic surgeries are performed to prevent the risk of pathological fractures and to achieve better functional results [4, 8, 11, 13]. Shavlokhova et al. have compiled data from 23 patients with brown tumors in the maxilla and mandible in their published review of the literature. They have reported that surgical resection was applied to 16 out of 23 patients. They stated that the treatment of brown tumors depends on the extent of the tumor mass and the tumor mass may need to be surgically removed in patients with inadequate response to conservative therapy [8]. In the literature, there are also many case reports of patients with brown tumor who underwent orthopedic surgery due to pathological fracture [5, 29–31]. In our case series, orthopedic surgeries were performed on 5 patients due to pathological fractures. Orthopedic surgeries were also performed on 18 lesions due to the risk of pathological fractures, while other 42 lesions were followed conservatively.

The treatment methods for brown tumors are determined by the size and location of the lesions as well as the functional issues associated with them. There is no consensus on which lesions parathyroidectomy will be sufficient and which lesions may require orthopedic surgery [4, 11, 31]. There is no objective scoring system similar to the Mirel criteria available to guide us in determining appropriate treatment methods for brown tumors. Mirels criteria is a scoring system used to assess the risk of pathological fractures in patients with metastatic bone lesions. The categories found in the Mirel scoring, such as location, pain severity, and size of the lesion, can help us in making treatment decisions. These parameters used in metastatic bone tumors can also be used as guidance when making decisions for prophylactic orthopedic surgery prior to pathological fractures in brown tumors [31, 32]. When we examined the 18 lesions in our case series, which were operated on prophylactically to prevent pathological fractures, it was observed that a majority of them were localized in the lower extremity, the pain scores were moderate to severe, and the sizes of the lesions were more than 50% of the bone diameter. There is a risk of pathological fracture in brown tumor cases that are followed conservatively without considering these criteria. A patient with a brown tumor in the femoral neck region underwent parathyroidectomy in an outside institution and was followed up conservatively. This patient was later admitted to our clinic due to a pathological fracture and total hip arthroplasty was performed.

If an orthopedic surgery is planned for a patient diagnosed with a Brown tumor, the timing of the surgery should be planned in coordination with the departments of anesthesiology, endocrinology, and general surgery. It should be remembered that hypercalcemia can lead to adverse intraoperative events. The patient should be referred to the endocrinology and general surgery departments for parathyroidectomy and calcium regulation. Hungry bone syndrome which is a medical condition characterized by rapid and prolonged hypocalcemia, may develop after parathyroidectomy. Until calcium regulation is achieved, definitive orthopedic surgery should be delayed [5, 33]. On the other hand, there are some studies have reported that orthopedic fixation was performed before the parathyroidectomy. In our case series, only 2 patients underwent orthopedic surgery before parathyroidectomy due to pathological fracture in the femoral diaphysis. In all other cases, parathyroidectomy was performed primarily, and orthopedic treatment was planned after calcium regulation was achieved.

Our study covers a wide time period spanning over 34 years, and it has been observed that there are some differences in the surgical techniques applied in different time periods. While the Kuntscher nail was applied to some patients with pathological fractures or fracture risk in the femur in the early 90s, with the advancements in orthopedic implant technology, the Kuntscher nail has been almost completely replaced by the new generation interlocking intramedullary nails. The methods preferred to fill the defect after curettage also varied among patients. In most cases, autograft was primarily preferred to fill the defect. In cases where the defect volume was large, allograft was also used in addition to autograft. We observed that in some cases where the defect volume was very large and allograft procurement was not possible, cementing had to be preferred. Although surgical methods and orthopedic implant materials may vary depending on time and the patient, the basic logic of the treatment remains the same. While parathyroidectomy is sufficient for some lesions, orthopedic surgeries are required for other lesions to prevent the risk of pathological fractures and achieve improved functional outcomes.

This study is limited by its retrospective design. As it was a retrospective study, it has not been possible to develop a new scoring system to guide brown tumor treatment. The criteria defined by Mirel for the prophylactic treatment of metastatic bone lesions were created as a result of a large-scale prospective study [32]. Due to the limited number of participants and the retrospective design of our study, the predictive value of Mirel scoring for pathological fractures in brown tumors could not be calculated. Another limitation of our study is the short follow-up period. Therefore, detailed analysis of long-term outcomes of lesions followed by conservative treatment could not be performed. A larger study with a prospective design and a longer follow-up period would be more informative. However, this paper is among the largest series on brown tumors in a single institute and provides important information about diagnosis and treatment.

## Conclusion

The diagnosis of brown tumor begins with clinical suspicion. When dealing with bone lesions, brown tumors and hyperparathyroidism should always be considered in the differential diagnosis. Biochemical analyses and the measuring PTH levels play a critical role in diagnosis. There is no consensus for the treatment algorithm. Pain intensity, location and size of the lesion are effective in determining the treatment method. While parathyroidectomy is sufficient for some lesions, orthopedic surgeries are required for some other lesions. Anesthesiology, endocrinology, and general surgery consultation is important before surgery. Treatment of brown tumors requires a multidisciplinary approach.
